# Financial toxicity in female patients with breast cancer: a national cross-sectional study in China

**DOI:** 10.1007/s00520-022-07264-3

**Published:** 2022-07-11

**Authors:** Meicen Liu, Linlin Hu, Xueyan Han, Man Cao, Jing Sun, Yuanli Liu

**Affiliations:** 1 School of Health Policy and Management, Chinese Academy of Medical Sciences and Peking Union Medical College, Beijing, 100730 People’s Republic of China; 2 Peking University First Hospital, Beijing, 100730 People’s Republic of China

**Keywords:** Financial toxicity, Coping strategy, Breast cancer, Female

## Abstract

**Purpose:**

To quantify financial toxicity of female patients with breast cancer in China and investigate its factors and patients’ coping strategies.

**Methods:**

The Comprehensive Score for Financial Toxicity (COST) is defined by using a structured questionnaire containing 12 items measuring perceived affordability of healthcare services, with the range of scoring of which being from 0 to 44 (higher score indicates lower financial toxicity). From January to March 2021, a total of 664 female patients diagnosed with stage 0–IV breast cancer were recruited from 33 public tertiary cancer hospitals located in 31 provinces of China. Multivariate linear regression models were used.

**Results:**

The median age of patients was 48 years (range: 26–84 years), and 62.04% lived in urban areas. The median COST score was 21.00 (interquartile range: 15–26). Older age, higher household income, and better self-reported health status were associated with lower financial toxicity, while a bigger household size, being retired or unemployed, stage IV cancer, and a history of targeted therapy were associated with higher financial toxicity (all *P* < 0.05). Nearly half of the patients reported using at least one coping strategy, including considering quitting treatment, delaying treatment, and failing to take medicine or attend medical visits as instructed. The people with increased financial toxicity seem to adopt more coping strategies.

**Conclusions:**

Financial toxicity and coping strategies are common among Chinese women with breast cancer. An understanding of the factors regarding financial toxicity may help oncologists and policy-makers identify at-risk patients and develop targeted interventions.

**Supplementary Information:**

The online version contains supplementary material available at 10.1007/s00520-022-07264-3.

## Introduction

In 2020, female breast cancer surpassed lung cancer as the leading cause of global cancer incidence, and it is the most frequently diagnosed cancer among Chinese women with more than 800 new cases each day [1, 2]. Due to the development of innovative treatments involving gene therapy, targeted therapy, and conservative surgery and the advances of overall healthcare, the health outcomes of patients with breast cancer have improved; however, treatment-related costs continue to increase [3]. A multicenter cross-sectional study including patients from 13 provinces in China reported that the average medical expenditure for a female patient with breast cancer was US $7,527 in 2014, which was more than twice the amount of the average disposable income of Chinese residents (US $3,284) [4]. Although social medical insurance of universal coverage has been built in China since 2009, the proportion of out-of-pocket expenditure in total health expenditure is still more than 25% now, with a higher proportion for cancer patients due to deficient coverage of certain drugs and procedures [5, 6]. The incidence of catastrophic health expenditure (CHE) for breast cancer was 31% (set the threshold of CHE at 40% of average household income) [7]. In China, the onset age of breast cancer is low, and patients are likely to have late-stage cancer due to poor awareness of regular screening protocols [8]. These factors may aggravate the financial hardship of female patients with breast cancer in China.

The financial hardship associated with clinical treatments is gaining recognition [9]. Previous studies have suggested that financial hardship is related to a decrease in treatment adherence and health-related quality of life and an increase in adverse symptoms and depression [10–15]. As a diagnosis of cancer is typically followed by a reduced work schedule and expensive treatments, many individuals or families are forced to use passive coping strategies, such as reducing non-medical expenses and basic healthcare expenses and acquiring loans [16, 17]. As financial burdens and their adverse effects are being gradually recognized, intervention strategies have been proposed including improving transparency regarding the costs of treatment, enhancing cost-based communication between patients and oncologists, and offering financial assistance [18].

Financial toxicity refers to the subjective financial distress and objective financial burden of medical care [19]. To quantify financial toxicity, the Comprehensive Score for Financial Toxicity (COST) instrument was developed [20, 21] and has been used and validated in several countries. Financial toxicity in patients with cancer has been associated with age [22, 23], income [24, 25], insurance [16], education [23, 24], out of pocket expenses [16], work status [21, 26], household savings [26], and disease and treatment profiles [16, 27]. The only previous study that investigated financial toxicity in Chinese female patients with breast cancer focused on patients with stage 0–III breast cancer treated at one tertiary cancer center [25]. More studies including a more representative sample are needed to further the understanding of this field.

This study aimed to quantify the financial toxicity of stage 0–IV breast cancer in female patients in China using the validated COST instrument and to explore the factors underlying high financial toxicity. The relationships between financial toxicity and coping strategies are also investigated in this study. By identifying the population most at risk for financial toxicity, this study may lead to policy changes and early interventions for those in need.

## Materials and methods

### Study design

This cross-sectional study based on a national inpatient survey was part of the external evaluation of the National Healthcare Improvement Initiative in 2021 and included patients from 33 public tertiary cancer hospitals in 31 provinces of China. The cancer hospitals included in this study treat a large number of patients with severe and complicated diseases and represent a relatively high technical level of diagnoses and treatments in China.

At least 150 inpatients diagnosed with any type and any stage of cancer were continuously recruited from each hospital between January and March 2021. Each patient was interviewed by the investigators. All of the patients were nearing discharge at the time of the interview, and those who agreed to participate in the study were asked to complete an electronic questionnaire. The investigators assisted patients who were unable to complete the questionnaire on their own. This study was approved by the Ethics Committee of Institute of Medical Biology of Chinese Academy of Medical Sciences (IPB-2020–23) and all patients provided written informed consent for their participation in the study.

### Patients

**Fig. 1:**
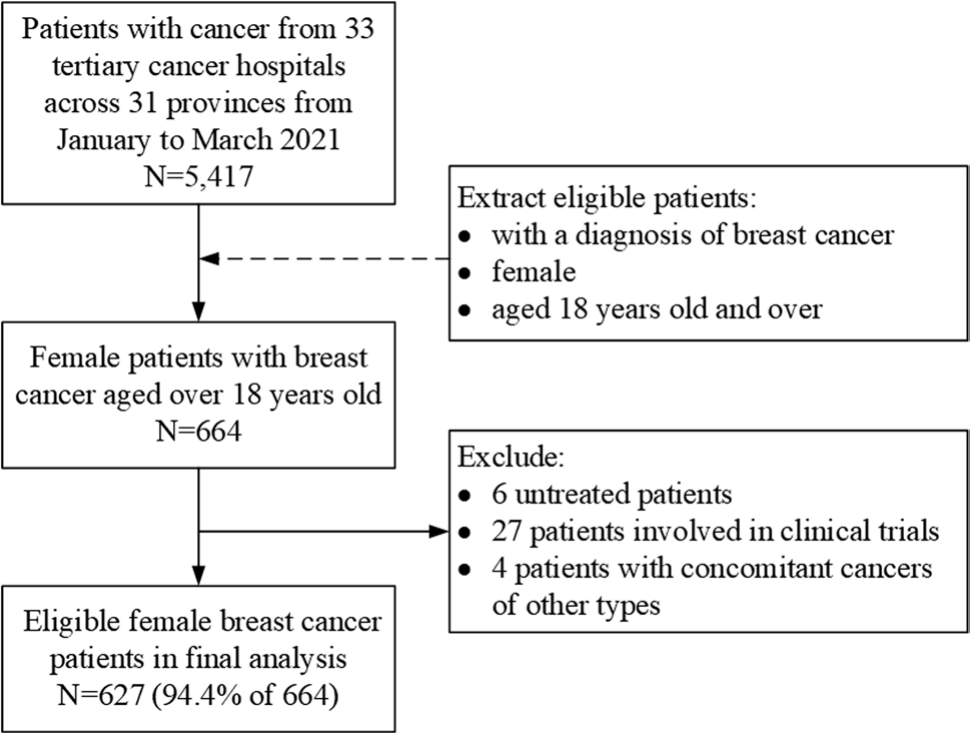
Flowchart of study population

A total of 5417 patients were surveyed. Female patients aged ≥ 18 years with a diagnosis of breast cancer (*n* = 664; 12.3%) were eligible for inclusion in this study. Patients who did not undergo treatment (*n* = 6), those involved in clinical trials (*n* = 27), and those with concomitant cancers of different types (*n* = 4) were excluded from the study as the financial burden of these patients could vary significantly from those undergoing regular treatments for breast cancer. The final analysis included 627 patients (Fig. 1).

### Variables and outcomes

In this study, financial toxicity was assessed using the COST questionnaire, which has been validated and used internationally and in China [20, 21, 28–32]. The COST questionnaire consists of 12 items that are rated using a 5-point Likert scale. The recall period of the COST questionnaire is 7 days. It is designed to measure the financial toxicity of patients aged 18 years and older. The total score of the COST questionnaire ranges from 0 to 44 points, with lower scores indicating worse financial conditions and higher levels of financial toxicity. The original English version of the COST questionnaire and the scoring guidelines are available online (www.facit.org/measures/FACIT-COST). The Chinese version can be obtained from the official website upon request (Supplementary A1).

The coping strategies of patients facing financial toxicity were designed based on frameworks and previous studies [17, 18, 33, 34]. They were collected by a survey question “have you ever taken the following actions due to financial difficulties,” followed by seven items (response “yes” or “no”):

1. once considered quitting treatment;
2. have delayed treatment for more than seven days;
3. have failed to take medicine as instructed;
4. have failed to attend medical visits as instructed;
5. have reduced spending on leisure activities, such as shopping or travelling;
6. have reduced spending on basic health services, such as clinic visits or vaccinations;
7. have borrowed money or acquired a loan due to illness.

The patients’ demographic (age, residency, ethnicity, marital status, education, and household size), economic (annual household income, work status, and medical insurance), and clinical (duration since diagnosis, cancer stage, self-reported health status, and therapies) characteristics were collected using a study-specific questionnaire based on relevant surveys that was validated by consultations with multidisciplinary experts and a small-scale pilot survey [27, 35].

### Statistical analysis

Descriptive statistics were used to summarize the patients’ baseline characteristics (Table 1). Continuous variables were presented as median and interquartile range (IQR) or mean and standard deviation (SD). Categorical variables were presented as number and percentage. Significant covariates identified in a univariate linear regression analysis (*P* ≤ 0.10) and covariates identified by other studies to be of significance (age and surgery) were included in the multivariate linear regression analyses [36, 37]. All of the variance inflation factors (VIF) for the multivariate liner regression analysis were less than two.

**Table 1:** Patient characteristics

Characteristics	*n* (%)	COST score (mean, SD)
*Demographic characteristics*		
Age (years; median,range)	48 (26–84)	20.99 (9.323)
Residency		
Rural	238 (37.96)	18.72 (9.039)
Urban	389 (62.04)	22.37 (9.234)
Ethnicity		
Han nationality	582 (92.82)	21.09 (9.391)
Minority nationality	45 (7.18)	19.62 (8.367)
Marital status		
Married	581 (92.66)	21.09 (9.275)
Non-married	46 (7.45)	19.74 (9.925)
Education		
College (> 12 years)	200 (31.90)	24.36 (8.993)
High school (9–12 years)	136 (21.69)	19.74 (8.420)
Junior school (6–9 years)	187 (29.82)	19.30 (9.390)
Primary school or less (≤ 6 years)	104 (16.59)	19.17 (9.337)
Household size		
1–3	345 (55.02)	22.25 (9.236)
≥ 4	282 (44.98)	19.45 (9.213)
*Economic characteristics*		
Annual household income (10 thousand yuan)^†^		
< 3	165 (26.32)	17.88 (9.153)
3–6	211 (33.65)	18.74 (8.367)
6–12	134 (21.37)	22.81 (9.177)
≥ 12	117 (18.66)	27.31 (7.761)
Work status		
Employed	439 (70.02)	21.77 (9.44)
Retired/unemployed	188 (29.98)	19.17 (8.792)
Medical insurance		
UEBMI	262 (42.79)	23.11 (9.207)
URBMI	338 (53.91)	19.17 (8.981)
Other	27 (4.31)	23.19 (10.149)
*Clinical characteristics*		
Duration since diagnosis (years)		
< 1	306 (48.80)	22.43 (9.317)
1–2	134 (21.37)	20.49 (9.039)
≥ 2	172 (27.43)	18.78 (9.025)
NA	15 (2.39)	21.33 (8.749)
Stage		
0–1	130 (20.73)	24.00 (8.394)
2	126 (20.10)	23.36 (9.310)
3	88 (14.04)	21.35 (10.038)
4	116 (18.50)	16.47 (8.523)
NA	167 (26.63)	19.80 (8.802)
Self-reported health		
Worse (0–60)	187 (29.82)	18.71 (9.105)
Moderate (61–80)	262 (41.79)	21.15 (8.929)
Better (81–100)	178 (28.39)	23.15 (9.618)
History of surgery		
No	136 (21.69)	20.54 (9.425)
Yes	491 (78.31)	21.11 (9.300)
History of targeted therapy		
No	497 (79.27)	21.67 (9.212)
Yes	130 (20.73)	18.36 (9.314)
History of radiotherapy		
No	445 (70.97)	21.85 (9.396)
Yes	182 (29.03)	18.88 (8.819)

*UEBMI*, Urban Employees Basic Medical Insurance; *URBMI*, Urban and Rural Residents Basic Medical Insurance; Other include commercial insurance, medical aid, and no medical insurance; *NA*, not available; *SD*, standard deviation. ^†^1.00 Chinese Yuan was equivalent to 0.15 US Dollar in 2021

In analysis of coping strategies, patients were divided into different financial toxicity groups based on median COST score [16, 25]. The patients’ coping strategies were presented as a number and percentage in each financial toxicity group. Pearson’s chi-square test was used to compare the coping strategies between the two groups with different COST scores.

For all analyses, a two-tailed *P* value < 0.05 was considered statistically significant. All statistical analyses were conducted with Stata/SE 15.0 software (Stata Corp LP, College Station, TX, USA).

### Sensitivity analysis

To verify the robustness of the result of determinants of financial toxicity, sensitivity analysis was performed by repeating the multivariate linear regression analysis with participants of a known cancer stage.

## Results

### Patient characteristics

The mean COST score was 20.99 (SD: 9.323) and the median COST score was 21.00 (IQR: 15–26). The mean patient age was 48.71 years (SD: 10.591 years), and 262 patients (42.79%) had Urban Employees Basic Medical Insurance (UEBMI) while 338 patients (53.91%) had Urban and Rural Residents Basic Medical Insurance (URBMI) (Table 1). Nearly half of the patients (48.80%) had a duration since diagnosis of less than one year. Stage 0–I cancer was diagnosed in 20.73% of patients, stage II in 20.10%, stage III in 14.04%, and stage IV in 18.50%. The stage was unknown in 167 (26.63%) patients. Overall, 78.31% of patients underwent surgical treatment, 20.73% underwent targeted therapy, and 29.03% underwent radiotherapy.

### Determinants of financial toxicity

The patients’ residency, education, household size, annual household income, work status, medical insurance, duration since diagnosis, stage, self-reported health status, history of targeted therapy, and history of radiotherapy were associated with financial toxicity in univariate analyses (all *P* < 0.05) (Table 2).

**Table 2:** Linear regression analysis of the financial toxicity

Patient characteristic	Univariate analysis		Multivariate analysis	
	Coefficient (95% *CI*)	*P* value	Coefficient (95% *CI*)	*P* value
Age	0.02 (− 0.05, 0.09)	0.606	0.10 (0.03, 0.17)	**0.004**
Residency				
Rural	ref		ref	
Urban	3.65 (2.17, 5.13)	** < 0.001**	0.48 (− 1.19, 2.16)	0.572
Education				
College (> 12 years)	ref		ref	
High school (9–12 years)	− 4.62 (− 6.60, − 2.65)	** < 0.001**	− 1.13 (− 3.18, 0.92)	0.280
Junior school (6–9 years)	− 5.06 (− 6.87, − 3.25)	** < 0.001**	− 1.61 (− 3.67, 0.44)	0.124
Primary school or less (≤ 6 years)	− 5.19 (− 7.34, − 3.04)	** < 0.001**	− 0.94 (− 3.58, 1.70)	0.486
Household size				
1–3	ref		ref	
≥ 4	− 2.80 (− 4.25, − 1.35)	** < 0.001**	− 1.45 (− 2.81, − 0.09)	**0.037**
Annual household income (10 thousand yuan)^†^				
< 3	ref		ref	
3–6	0.86 (− 0.91, 2.62)	0.340	0.03 (− 1.67, 1.72)	0.976
6–12	4.93 (2.95, 6.90)	** < 0.001**	3.36 (1.38, 5.34)	**0.001**
≥ 12	9.43 (7.38, 11.48)	** < 0.001**	6.30 (4.03, 8.57)	** < 0.001**
Work status				
Employed	ref		ref	
Retired/unemployed	− 2.60 (− 4.18, − 1.01)	**0.001**	− 2.08 (− 3.57, − 0.59)	**0.006**
Medical insurance				
UEBMI	ref		ref	
URBMI	− 3.94 (− 5.41, − 2.46)	** < 0.001**	− 1.48 (− 3.16, 0.20)	0.083
Other	0.08 (− 3.54, 3.70)	0.966	1.05 (− 2.20, 4.31)	0.525
Duration since diagnosis (years)				
< 1	ref		ref	
1–2	− 1.94 (− 3.82, − 0.7)	**0.042**	− 1.63 (− 3.33, 0.07)	0.061
≥ 2	− 3.65 (− 5.37, − 1.93)	** < 0.001**	− 1.49 (− 3.25, 0.26)	0.095
NA	− 1.30 (− 6.09, 3.48)	0.594	− 2.19 (− 6.48, 2.09)	0.315
Stage				
0–I	ref		ref	
II	− 0.64 (− 2.84, 1.56)	0.566	1.03 (− 1.01, 3.08)	0.320
III	− 2.65 (− 5.08, − 0.22)	**0.033**	− 0.13 (− 2.47, 2.20)	0.912
IV	− 7.53 (− 9.78, − 5.29)	** < 0.001**	− 4.35 (− 6.64, − 2.06)	** < 0.001**
NA	− 4.20 (− 6.25, − 2.14)	** < 0.001**	− 2.28 (− 4.23, − 0.33)	**0.022**
Self-reported health				
Worse (0–60)	ref		ref	
Moderate (61–80)	2.44 (0.72, 4.17)	**0.006**	1.72 (0.16, 3.28)	**0.031**
Better (81–100)	4.44 (2.55, 6.33)	** < 0.001**	3.77 (2.06, 5.48)	** < 0.001**
History of surgery				
No	ref		ref	
Yes	0.56 (− 1.20, 2.35)	0.525	− 0.66 (− 2.27, 0.94)	0.417
History of targeted therapy				
No	ref		ref	
Yes	− 3.31 (− 5.10, − 1.53)	** < 0.001**	− 2.36 (− 3.98, − 0.74)	**0.004**
History of radiotherapy				
No	ref		ref	
Yes	− 2.96 (− 4.56, − 1.37)	** < 0.001**	− 0.64 (− 2.21, 0.93)	0.422

Bold *P* values < 0.05. A negative value indicates a lower score and higher financial toxicity compared with reference. ^†^1.00 Chinese Yuan was equivalent to 0.15 US Dollar in 2021

*UEBMI*, Urban Employees Basic Medical Insurance; *URRMI*, Urban and Rural Residents Basic Medical Insurance; Other include commercial insurance, medical aid, and no medical insurance; *NA*, not available

After adjusting for possible confounding variables, older age, higher annual household income, and better self-reported health status were associated with lower financial toxicity (all *P* < 0.05) (Table 2). Per year increase in age was associated with an average increase in the COST score of 0.10 points (95% confidence interval (CI): 0.03 to 0.17, *P* = 0.004). Annual household income of ¥60,000–120,000 and ≥ ¥120,000 increased the COST score by 3.36 points (95% CI: 1.38 to 5.34, *P* = 0.001) and 6.30 points (95% CI: 4.03 to 8.57, *P* < 0.001), respectively, compared to an annual household income < ¥30,000. Self-reported health status of moderate or better were associated with COST scores that were increased by 1.72 points (95% CI: 0.16 to 3.28, *P* = 0.031) and 3.77 points (95% CI: 2.06 to 5.48, *P* < 0.001), respectively.

Smaller household size, being retired or unemployed, advanced stage, and having a history of targeted therapy were associated with lower COST scores (higher financial toxicity). A household size ≥ 4 was associated with an average 1.45-point (95% CI: − 2.81 to − 0.09, *P* = 0.037) decrease in the COST score. The average COST score of patients who were retired or unemployed was 2.08 points (95% CI: − 3.57 to − 0.59, *P* = 0.006) lower than that of patients who were employed. Patients with stage IV breast cancer scored 4.35 points (95% CI: − 6.64 to − 2.06, *P* < 0.001) lower than patients with stage 0–I breast cancer, and those who did not know their stage scored 2.28 points (95% CI: − 4.23 to − 0.33, *P* = 0.022) lower than patients with stage 0–I breast cancer. Patients who underwent targeted therapy averaged 2.36 points (95% CI: − 3.98 to − 0.74, *P* = 0.004) lower than those who had not. Patients with URBMI were more likely to have increased financial toxicity risk than those with UEBMI (coefficient: − 1.48, 95% CI: − 3.16 to 0.20, *P* = 0.083). Patients whose duration since diagnosis was 1–2 years (coefficient: − 1.63, 95% CI: − 3.33 to 0.07, *P* = 0.061) or ≥ 2 years (coefficient: − 1.49, 95% CI: − 3.25 to 0.26, *P* = 0.095) had increased financial toxicity compared with those whose duration since diagnosis was ≤ 1 year (Table 2).

### Patients’ coping strategies and financial toxicity

Nearly half of the patients (48.01%) reported using at least one coping strategy (Table 3). Overall, 258 (41.15%) patients reported decreasing spending on leisure activities such as shopping or travelling, and 106 (16.91%) reported decreasing their spending on basic health services such as clinic visits and vaccinations. A total of 133 (21.21%) patients borrowed money from relatives and friends or acquired a loan from a bank. Patients with COST scores below the median score (those with high financial toxicity) were more likely to use coping strategies (all *P* < 0.001).

**Table 3:** The frequency and percentage of patients taking coping strategies by COST score group

Items	Entire cohort (*n* = 627) *N* (%)	COST score < 21 (*n* = 300) *N* (%)	COST score ≥ 21 (*n* = 327) *N* (%)
Considered quitting treatment	73 (11.64)	57 (19.00)	16 (4.89)
Delayed treatment for more than seven days	34 (5.42)	26 (8.67)	8 (2.45)
Failed to take medicine as instructed	42 (6.70)	31 (10.33)	11 (3.36)
Failed to attend medical visits as instructed	21 (3.35)	17 (5.67)	4 (1.22)
Reduced spending on leisure activities, such as shopping or travelling	258 (41.15)	172 (57.33)	86 (26.30)
Reduced spending on basic health services, such as clinic visits or vaccinations	106 (16.91)	69 (23.00)	37 (11.31)
Borrowed money or acquired a loan due to illness	133 (21.21)	116 (38.67)	17 (5.20)
At least one coping strategy above	301 (48.01)	204 (68.00)	97 (29.66)

**Fig. 2:**
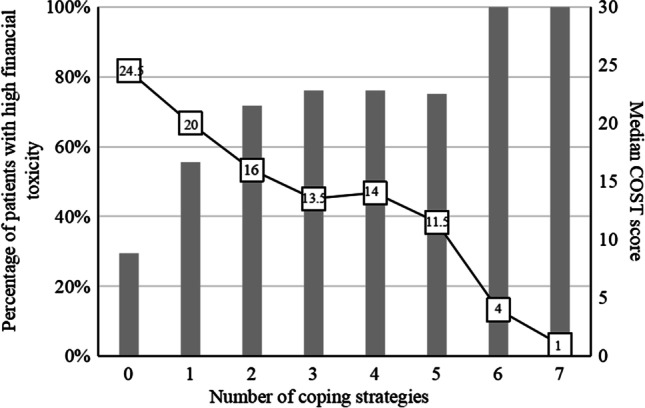
Financial toxicity by coping strategies

As the number of coping strategies used by a patient increased from zero to seven, the percentage of patients with high financial toxicity increased from 29.5 to 100.0% (Fig. 2). The median COST score decreased as the number of coping strategies used increased, as the median COST score for patients using zero coping strategies was 24.5 (IQR: 20–31) and that of patients using seven coping strategies was 1 (IQR: 0–4).

### Sensitivity analysis

A total of 460 patients knew their cancer stage. A regression analysis including these patients yielded similar results as the analysis including all patients (Supplementary A2).

## Discussion

This study identified factors and coping strategies associated with financial toxicity in female patients with breast cancer using the COST score. Older age, higher household income, and better self-reported health status were associated with lower financial toxicity, while bigger household size, being retired or unemployed status, stage IV breast cancer, and a history of targeted therapy were associated with higher financial toxicity.

### The level of financial toxicity of patients with breast cancer in China

The multicenter design of this study allowed for the observation of financial toxicity in a more geographically and clinically diverse population in China. A previous study reported financial toxicity of 166 patients with stage 0–III breast cancer treated at a single hospital [25]. Our study includes patients with stage 0–IV breast cancer patients treated at 33 hospitals. The median COST score was a little lower in this study than the score of 22 in the previous study [25], which may be attributed to the inclusion of patients with stage IV breast cancer in this study (stage IV: 18.50%, median COST score: 17). The median COST score reported in a study of financial toxicity in patients with breast cancer who underwent lumpectomy or mastectomy in the USA was 30 and the median COST score reported in patients with metastatic breast cancer was 22, which are higher than in our study [16, 38]. The comparatively higher level of financial toxicity among Chinese patients might be attributed to inadequate coverage of medical insurance for cancer treatment cost and the low affordability of advanced drugs and procedures given that China is still a middle-income country with a big population. [39, 40]. Besides, in the USA, median scores of financial toxicity among cancer survivors were mostly between 23 and 29, and financial toxicity of patients with breast cancer was relatively lower than patients with other types of cancer, such as multiple myeloma and lung cancer due to lower medical costs [21, 23, 27]. These indicate greater economic burden for Chinese cancer survivors.

### Factors associated with financial toxicity

In this study, age and household size were associated with financial toxicity. Patients with increased age had a lower probability of having financial toxicity, which is consistent with the results of previous studies [22, 41–43]. Younger adults may not have additional resources such as retirement funds or home equity to ease their financial burden [44]. Younger adults also tend to have fewer savings, and an association between less household savings and higher financial toxicity has been reported [11, 26]. Younger adults also have more financial responsibilities than older adults, including the need to support family members and pay for housing and other bills, resulting in increased financial needs [45]. In this study, a larger household size was associated with higher financial toxicity in the multivariate models among patients with breast cancer, which is consistent with the findings of previous studies [41, 46, 47].

Economic factors are important predictors of financial burden for patients with cancer [47]. In this study, household income was a protective factor of financial toxicity. Previous studies reported an association between being retired or unemployed and higher financial toxicity [21, 47] Different medical insurance types showed no association with financial toxicity. Chinese government launched a new round of health system reform in 2009 to provide affordable and equitable basic healthcare for all, the gap of different medical insurance types regarding service packages covered and the drugs covered was greatly narrowed [5].

The effects of disease duration, disease stage, self-reported health status, and treatment methods (including surgery, radiotherapy, and targeted therapy) on financial toxicity were also investigated in this study. Better self-reported heath status was a protective factor for patients in this study. A previous study regarding the correlation between COST scores and self-reported health in gynecologic oncology patients reported that worse self-reported health is correlated with greater financial toxicity (*r* = 0.47; *P* < 0.001) [34]. Advanced cancer stage was significantly associated with increased financial toxicity in this study, which is similar to previously reported results regarding financial toxicity in adult females with breast cancer who underwent a lumpectomy or mastectomy [16]. Increased financial toxicity was also observed among patients with an unknown breast cancer stage. In China, a patient’s family tended to conceal the details of the disease from the patient, especially when the disease is severe [48, 49]. Therefore, patients who do not know their stage may have a higher probability to suffer advanced stage cancer. A history of targeted therapy increased the risk of financial toxicity in this study. These findings indicate that multiple policies or actions should be taken by the government and society to relieve the financial difficulties faced by patients with breast cancer, especially those who are younger adults, unemployed, suitable for targeted therapy, and with bigger household size, lower income and worse self-reported health.

### Financial toxicity and coping strategies

Increased financial toxicity resulted in the use of multiple coping strategies, as indicated by the association between decreased COST scores and increased number of coping strategies. A previous study reported that patients prioritized affordability or maintaining functional independence when making treatment decisions [44]. Sixty-eight percent of patients with high financial toxicity and nearly 30% of patients with low financial toxicity reporting using at least one coping strategy, which includes reducing their spending on basic health services such as clinic visits and vaccinations, quitting treatment, delaying treatment, and failing to take medicine or attend medical visits as instructed. These results indicate patients with financial difficulties tend to withdraw from treatment plans or avoid seeking necessary healthcare, which might cause adverse effects on their recovery and health outcome [15].

### Strengths and limitations

Our study was based on a large sample with a diversity of geographic locations and multicenter collaboration in China. Our findings identified the common coping strategies of patients with breast cancer who faced financial toxicity. Patients with higher financial toxicity tended to pause or postpone appropriate treatments and reduce their basic healthcare and basic living expenses. The results of this study can help identify patients with a high-risk financial toxicity, and are useful for designing targeted interventions.

However, this study has some limitations. First, the nature of cross-sectional design limited the power to determine a causal relationship. Longitudinal research will be considered to explore the causal mechanism in the future. Second, the clinical information was self-reported by the patients which may have recall bias, although the magnitude of the bias might be small because the patients were interviewed in hospitals close to discharge. In addition, approximately one-fourth of the patients in this study did not know their cancer stage. A sensitivity analysis was used to assess whether the exclusion of this population would significantly affect the results. And results of the sensitivity analysis were robust. Third, travel distance between home and treatment center is an important factor related to indirect costs and financial toxicity. Our survey did not collect the data and it could be further explored in future studies. Finally, this study only focused on female patients with breast cancer in tertiary cancer hospitals, which usually took patients of progressive stage. Future studies should include more hospital and disease types.

## Conclusion

In conclusion, this national cross-sectional study that quantified financial toxicity in female patients with stage 0–IV breast cancer in China revealed that financial toxicity and coping strategies are common among these patients. Increased financial toxicity is associated with the use of more coping strategies. An understanding of the factors associated with financial toxicity may help oncologists and policy-makers identify patients at risk for financial toxicity and develop targeted interventions.

## Supplementary Information

Below is the link to the electronic supplementary material.

Supplementary file1 (DOC 18 KB)

Supplementary file2 (PDF 231 KB)

## Data Availability

The datasets generated during and/or analyzed during the current study are available from the corresponding author on reasonable request.
