# Supplementary material for: Financial toxicity in female patients with breast cancer: a national cross-sectional study in China
Source: Support Care Cancer. 2022 Jul 11;30(10):8231–40. doi: 10.1007/s00520-022-07264-3 (PMC9512750; doi:10.1007/s00520-022-07264-3)
Supplement: Supplementary file 1 — Supplementary file1 (DOC 18 KB) [file 520_2022_7264_MOESM1_ESM.doc]

**Supplementary A1. Chinese version of COST questionnaire.**

COST–FACIT (Version 2)

以下是一些与您患有同样疾病的人所认为重要的陈述。请在每行圈选或标出一个数字来表明适用于您过去7天情况的回答。

**FT1 我知道我有足够的存款、退休金或者资产来支付我的治疗费用**

0-一点也不 1-有一点 2-有些 3-相当 4-非常

**FT2 我自掏腰包的医疗花费比我想象的多**

0-一点也不 1-有一点 2-有些 3-相当 4-非常

**FT3 因为我的疾病或治疗，所以我担心我将来会有经济问题**

0-一点也不 1-有一点 2-有些 3-相当 4-非常

**FT4 我感觉我对花在照护上的费用别无选择**

0-一点也不 1-有一点 2-有些 3-相当 4-非常

**FT5 我不能像往常那样工作或贡献，让我沮丧**

0-一点也不 1-有一点 2-有些 3-相当 4-非常

**FT6 我对我目前的经济状况很满意**

0-一点也不 1-有一点 2-有些 3-相当 4-非常

**FT7 我能够承担我每个月的开支**

0-一点也不 1-有一点 2-有些 3-相当 4-非常

**FT8 我感觉经济上有压力**

0-一点也不 1-有一点 2-有些 3-相当 4-非常

**FT9 我担忧是否还能继续保持我的工作和收入，包括在家工作**

0-一点也不 1-有一点 2-有些 3-相当 4-非常

**FT10 癌症或治疗降低了我对自己现在经济状况的满意度**

0-一点也不 1-有一点 2-有些 3-相当 4-非常

**FT11 我感觉我能掌控我的经济状况**

0-一点也不 1-有一点 2-有些 3-相当 4-非常

**FT12 我的疾病给我和我的家庭带来了经济困难**

1. 一点也不 1-有一点 2-有些 3-相当 4-非常
