# Supplementary figures and images for: Financial toxicity in female patients with breast cancer: a national cross-sectional study in China
Source: Support Care Cancer. 2022 Jul 11;30(10):8231–40. doi: 10.1007/s00520-022-07264-3 (PMC9512750; doi:10.1007/s00520-022-07264-3)

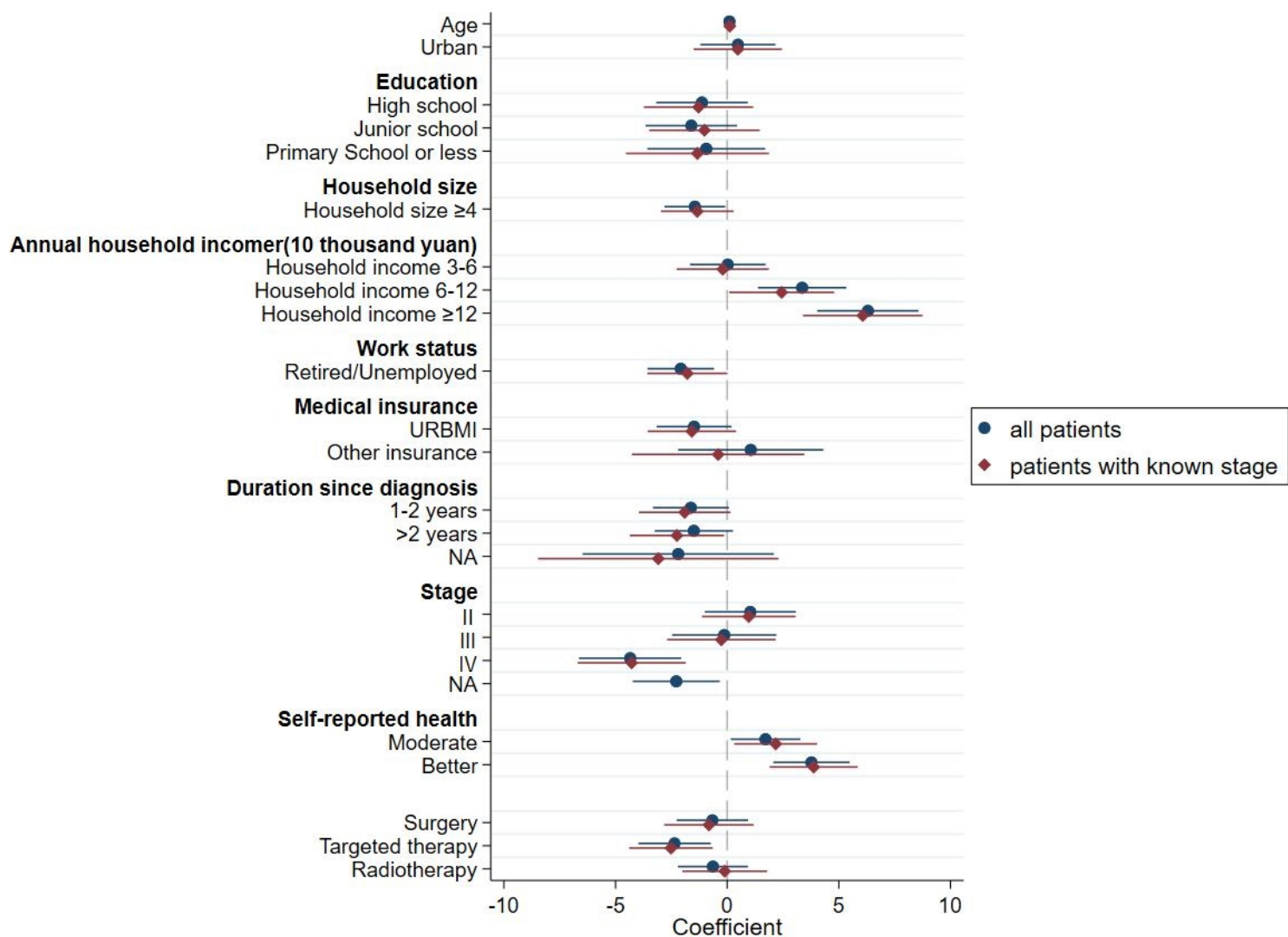

Supplement: Supplementary file 2 — Supplementary file2 (PDF 231 KB) [file 520_2022_7264_MOESM2_ESM.pdf]
